# Environmental Exposure to Non-Persistent Endocrine Disrupting Chemicals and Endometriosis: A Systematic Review

**DOI:** 10.3390/ijerph19095608

**Published:** 2022-05-05

**Authors:** Katarzyna Wieczorek, Dorota Szczęsna, Joanna Jurewicz

**Affiliations:** Department of Chemical Safety, Nofer Institute of Occupational Medicine, 91-348 Lodz, Poland; dorota.szczesna@imp.lodz.pl (D.S.); joanna.jurewicz@imp.lodz.pl (J.J.)

**Keywords:** environmental exposure, non-persistent endocrine-disrupting chemicals, endometriosis, phthalates, bisphenol A, parabens, benzophenones

## Abstract

Endometriosis is a disease characterized by the presence of the uterine endometrium outside of its normal location. As the etiology of endometriosis is not well known and hormonal imbalance is central to disease pathogenesis, the potential contribution of exposure to endocrine-disrupting chemicals (EDCs) has been hypothesized in endometriosis. A systematic search of the literature was carried out to identify relevant studies using: PubMed, Scopus, Elsevier, Springer; EBSCO, and Web of Science. A total of 22 studies were considered. Most of the studies reviewed in this paper showed an association between exposure to BPA and phthalates and endometriosis. In the case of phthalate exposure, the reviewed studies found an association between the concentration of at least one phthalate metabolite and endometriosis. Only one study was performed to assess the exposure to parabens and a significant relationship with endometriosis was found. Additionally, only one study assessed the relationship of non-persistent pesticide exposure with endometriosis, observing a significant association between endometriosis and the urinary concentration of diazinon, chlorpyrifos, and chlorpyrifos-methyl. Studies struggled to provide a conclusion on the effect of exposure to benzophenones on endometriosis. Despite the numerous limitations of the results, the reviewed studies suggest that exposure to non-persistent endocrine disruptors, especially bisphenol A and phthalates may affect endometriosis. The results of the studies on exposure to parabens, benzophenones, and non-persistent insecticides are inconclusive.

## 1. Introduction

Endometriosis is a gynecological disease associated with chronic pelvic pain and infertility. This disease is diagnosed in 5–10% of women of reproductive age [1]. For a woman, the disease is associated with malaise, undergoing therapy, and performing costly operations. Women often have to give up their daily duties because of the bothersome symptoms of the disease. Endometriosis is characterized by the presence of uterine endometrial tissue outside of its normal location. Pathological endometrial tissue occurs on the peritoneum of the pelvis, ovaries, and rectovaginal septum, as well as even in the pericardium, pleura, and brain. The disease causes extensive adhesions and distortions in the pelvis [2]. Endometriosis is diagnosed surgically using the technique of laparoscopy or laparotomy [3]. Another method of endometriosis diagnosis is vaginal ultrasound (TVUS) or magnetic resonance imaging (MRI) [4,5]. The American Society for Reproductive Medicine (ASRM) guidelines are used to assess the severity of the disease. There are four degrees of endometriosis: minimal, mild, moderate, and severe [6]. In addition, there are three types of endometriosis: peritoneal, ovarian, and rectovaginal. The clinical picture differs depending on the patient, and the treatment procedures depend on the symptoms and fertility status [7]. Endometriosis is idiopathic in origin, but there are several theories that could explain its origin. It is assumed that the following factors may be responsible for the development of endometriosis: immune dysfunction, genetic aspects, lifestyle, and environmental pollution [8,9,10]. As the etiology of endometriosis is not well understood and estrogen is central to disease pathogenesis, regulating the key pathological processes in endometriosis including immunological, inflammatory, angiogenic, antiapoptotic, cellular, and molecular mechanisms, the potential contribution of exposure to endocrine-disrupting chemicals (EDCs) has been hypothesized in endometriosis. EDCs are of particular interest as potential contributors to endometriosis because they can alter steroidogenesis and immunologic function, in addition to being the epigenetic causal factors involved in disease progression [11]. According to WHO’s definition, an endocrine disruptor is an exogenous substance or mixture that alters the function(s) of the endocrine system and consequently causes adverse health effects in an intact organism, its progeny, or its subpopulations (WHO 1996). EDCs are compounds that have the ability to interact with the endocrine system interfering with its normal functioning. They are widespread in the environment; thus, exposure occurs through contact with these compounds through food, water, air, plastics, or cosmetics. EDCs can be categorized as persistent or non-persistent. Persistent chemicals are those chemicals that tend to endure in the environment for years after their release, whereas, nonpersistent chemicals are exogenous chemicals or mixtures of industrial agents that can interfere with the normal action of hormones with a shorter half-life and lower liposolubility [11].

Numerous studies have been conducted on the influence of exposure to persistent endocrine-disrupting factors (e.g., dioxins, polychlorinated biphenyls, organochlorine pesticides, and some metals) on endometriosis. The obtained results did not confirm the validity of the statement concerning the influence of exposure to dioxins on the risk of developing endometriosis [12,13]. Similarly, numerous studies have been carried out on the influence of polychlorinated biphenyls on the occurrence of endometriosis, and most of them failed to confirm the existence of associations between these chemicals and the occurrence of endometriosis [12,14,15,16]. In addition, studies on persistent pesticide exposure and endometriosis have been conducted, but the results did not provide a clear indication of a link between pesticide exposure and the occurrence of endometriosis [17,18,19].

Non-persistent compounds are chemicals widely found in the environment. in many everyday products, e.g., plastics, lubricants, solvents, plasticizers, and pesticides. Low levels of exposure may cause endocrine or reproductive disorders [20]. These are substances that may disrupt the functioning of the endocrine system and, consequently, affect the fertility of men and women [21,22,23,24,25,26]. Compared to studies evaluating the link between persistent endocrine-disrupting chemical exposure and endometriosis studies on the effects of exposure to nonpersistent chemicals are rare. As the exposure to non-persistent chemicals is widespread and associated with reproductive and gynecological disorders, as well as poor fertility, this review aims to answer the question regarding the effect of exposure to widespread nonpersistent endocrine-disrupting chemicals on endometriosis, taking into account the limitations and strength of the presented studies.

## 2. Materials and Methods

The PRISMA (Preferred Reporting Items for Systematic Reviews and Meta-Analyses) guidelines was employed in this review.

### 2.1. Search Strategy

A systematic search of the literature was carried out to identify relevant studies published in English from 2003 to February 2021. Relevant studies were also identified through a review of the references cited in all the published studies. The following databases were used: PubMed, Scopus, Elsevier, Web of Science, Springer, and EBSCO. The keywords for our search included a combination of terms referring to exposure to non-persistent chemicals and outcome, i.e., exposure to bisphenols, benzophenones, phthalates, parabens, organophosphate pesticides, synthetic pyrethroids, and endometriosis.

The most recent human studies published in English in peer-reviewed journals since 2003 were included in this review.

The period was chosen to reflect findings over the past 19 years. During that time, animal and in vitro studies provided evidence on the toxicity of several non-persistent environmental EDCs, especially in the case of endometriosis risk.

In total, 482 articles were found as a result of the search, and they were all checked for eligibility. The reference lists of the selected articles were subject to a manual search to identify additional articles.

### 2.2. Selection Criteria

A total of 22 publications on exposure to non-persistent endocrine-disrupting chemicals and endometriosis were selected by two reviewers, with an excellent agreement (k = 0.80). This review included original peer-reviewed studies that looked at exposure to non-persistent endocrine-disrupting chemicals and endometriosis in humans. The majority of the articles (80%) did not meet the inclusion criteria for our study, as they did not address the endometriosis risk. Publications containing duplicate data or published before 2003 were excluded. We also excluded studies that analyzed the impact of environmental EDCs on different endocrinological disorders (uterine leiomyoma, polycystic ovary syndrome, and recurrent miscarriages), as well as occupational exposure studies. Articles focused on animal research, in vitro studies, and review papers as well as articles published in a language other than English were excluded.

### 2.3. Study Selection

Two researchers identified relevant articles and independently assessed those to be included in this review; incongruences were resolved by discussion and the intervention of a third independent author. Articles were displayed by title and abstract. Duplicate and irrelevant items were excluded. The remaining articles were subjected to a full-text review. All full-text articles were thoroughly examined to identify the aims of the studies, statistical methods, and accurate results. The following information was taken into account when selecting the studies: authors and years of publication; the main purpose of the study; results; type of study; accuracy of the results. For the purpose of this review, the following information was abstracted from each study: study population; type of exposure and methods used for its assessment (including biomarkers); type of study, level of exposure to selected endocrine-disrupting factor; results. All articles cited were summarized and discussed.

## 3. Results

### 3.1. Exposure to Phthalates and Endometriosis

Phthalates are synthetic chemicals with a wide range of applications. They are used in the production of plastics, e.g., paints, adhesives, floors, rubber materials, medicines, and even packages intended for contact with food [27,28,29,30]. Due to their wide application, they are present in the environment. Exposure to phthalates occurs through food, skin, and air [31,32]. Some phthalates can cause allergies and may disrupt the functioning of the human endocrine system [33,34,35]. Moreover, it has been found that exposure to phthalates during fetal life may affect the DNA methylation of genes responsible for the androgenic, estrogenic, and spermatogenetic responses [36]. Research has been conducted on the effects of phthalates on children’s health, reproductive disorders, and premature puberty among girls [37]. The studies showed an adverse effect of phthalates on the level of reproductive hormones such as luteinizing hormone, free testosterone, and sex hormone-binding globulin, as well as thyroid function [38,39].

In the human body, phthalates have a short half-life of about 12 h [40]. Phthalates can be divided into short branched and long-branched phthalates. Short-branched phthalates are hydrolyzed to monoester phthalates and then excreted in the urine. In contrast, long-branched phthalates undergo several biotransformations and are then excreted in the urine or feces.

The association between environmental exposure and the occurrence of endometriosis was investigated in 12 studies [41,42,43,44,45,46,47,48,49,50,51,52] (Table 1), including 9 case-control studies [41,42,43,44,45,47,49,50,51], 2 cross-sectional studies [46,52], and 1 cohort study [48]. The studies were performed in the United States, Brazil, India, Italy, Taiwan, Pakistan, Japan, Korea, and China. Women in most of the studies were recruited from fertility and gynecology centers [43,45,49,50,51], three studies were conducted in hospitals or universities [42,47,48], two were based on the National Health and Nutrition Examination Survey (NHANES) [46,52], and one was based on US health system research in the Pacific Northwest [41]. The concentrations of phthalates were mostly determined in urine samples [41,42,45,46,48,49,50,52], followed by serum samples [43,44,47,51] and peritoneal fluid [44]. The age of women participating in the studies ranged from 18 to 54 years. In studies performed by Upson et al. (2013) [41], Fernandez et al. (2019) [42], Reddy et al. (2006) [43], Weuve et al. (2010) [46], Louis et al. (2013) [48], Itoh et al. (2009) [49], Kim et al. (2015) [50], Kim et al. (2011) [51], and Zhang et al. (2021) [52], a questionnaire about women’s lifestyle factors (e.g., smoking, sporting activity, and birth control), was used, whereas in studies by Cobellis et al. (2003) [44], Huang et al. (2010) [45], and Nazir et al. (2018) [47], lifestyle factors were not assessed. Endometriosis in most studies was diagnosed by surgery or magnetic resonance imaging [41,42,43,44,45,47,48,49,50,51]. Endometriosis diagnosis was based on a questionnaire in two studies [46,52].

In the study by Upson et al. (2013) [41], there was a strong inverse relationship between urinary MEHP (mono-(2-ethyl)-hexyl phthalate) content and endometriosis (OR: 0.3, 95% CI: 0.1–0.7). In the study by Huang et al. (2010) [45], an increased concentration of a phthalate metabolite, MnBP (mono-benzyl phthalate) was observed in cases versus controls (OR: 3.46, 95% CI: 1.16–10.3). The study by Nair et al. (2018) [47] showed that exposure to DEHP (diethylhexyl phthalate) was only related to advanced stages of endometriosis (stage III and IV). Similar conclusions regarding the advanced stages of endometriosis and exposure to MEHP were obtained by Kim et al. (2011) [51]. Another study performed by Kim et al. (2015) [51] showed a statistically significant association between the concentration of three phthalate metabolites, MEHHP (mono-(2-ethyl-5-hydroxyhexyl) phthalate) (OR:2.52, 95% CI: 1.03–6.14), MEOHP (-mono-(2-ethyl-5- oxohexyl) phthalate) (OR:2.89, 95% CI: 1.04–8.04), and MECPP (mono-(2-ethyl-5-carboxypentyl) phthalate) (OR:2.57, 95% CI: 0.92–7.13) and endometriosis. Louis et al. (2013) [48] showed a significant relationship between the occurrence of endometriosis and exposure to phthalates (MBP, MCMHP, MECPP, MEHP, MEHHP, MEOHP, and MOP) in the population cohort group. In the study conducted by Reddy et al. (2006) [43], phthalates (DnBP, BBP, DnOP, and DEHP) were observed in all samples of women with endometriosis; moreover, the results for all samples were statistically significant at *p* < 0.05 compared to control women. Kim et al. (2015) [50] found that the urinary concentrations of MEHHP, MEOHP, and MECPP were significantly higher in women with endometriosis compared to controls.

In five studies, no association was found between the concentration of phthalates and the risk of endometriosis [42,44,46,49,52].

In conclusion, seven of the presented studies found an association between the concentration of at least one phthalate metabolite and endometriosis. In five studies, no association was noted between phthalate concentration and endometriosis.

### 3.2. Exposure to Bisphenol A and Endometriosis

Bisphenol A (BPA) is a monomer found in many plastics and epoxy resins. Bisphenol A is a common chemical in everyday items. Approximately eight billion pounds of BPA are produced annually, of which up to over 200 thousand pounds per year may be released into the environment [53]. BPA is used in the production of toys, containers for drinks and food, sports equipment, medical equipment, and cables. Human exposure occurs through the diet, through inhalation of house dust, and through the skin [54]. Bisphenol A has been shown to disrupt hormonal balance. This compound shows estrogenic activity because it binds to estrogen receptors [55]. Moreover, it has been shown that BPA can bind to androgens, blocking their endogenous action, and may it act on the secretion of thyroid hormones [56]. Exposure of the fetus and newborn to bisphenol A may have negative developmental effects such as reduced maturation cycle, prostate changes, altered development of mammary glands, changes in body weight, and changes in the brain. On the other hand, exposure to bisphenol A in adulthood may lead to sperm damage, a decrease in estradiol, miscarriage or premature birth, development of diabetes mellitus [57], and reproductive system impairment in both women and men [58]. Low doses of BPA may also affect the functioning of the human endocrine system [59].

The relationship between exposure to bisphenol A and the development of endometriosis was investigated in nine studies [42,48,49,60,61,62,63,64,65,66] (Table 2). Seven of them were performed using a case-control study design [42,60,61,62,63,64,65], while one was conducted as a cross-sectional study [66], and one was conducted as a cohort study [48]. The studies were performed in Brazil, Japan, the United States, Spain, Iran, Italy, and China. The majority of women in the studies were recruited from gynecological hospitals or fertility treatment centers [61,63,64,65,66], while the remainder were involved in studies conducted by hospitals or universities [42,48,62]. One study was conducted within the U.S. health system in the Pacific Northwest [60]. BPA concentrations were mostly analyzed mostly in urine (eight studies) [42,48,60,61,62,63,65], while only study evaluated serum concentrations [64]. Women aged 18 to 54 years were recruited for the studies [42,48,60,61,62,63,64,65,66], and they were diagnosed by surgery laparoscopy, laparotomy, or magnetic resonance imaging. In eight studies all women completed a questionnaire about lifestyle [42,48,60,61,62,63,65,66], while one study did not implement a questionnaire [64].

Upson et al. (2014) [60] found a significant association of exposure to BPA with the occurrence of non-ovarian pelvic endometriosis (OR:3.0; CI: 1.2, 7.3), but not with the occurrence of ovarian endometriosis. Peinado et al. (2020) [61] observed a relationship between BPA concentration and the occurrence of endometriosis (OR: 1.5, 95%; CI: 1.0–2.3). Rashidi et al. (2017) [62] revealed the relationship between BPA concentration in urine and the occurrence of endometriosis (OR: 1.75, 95%; CI: 1.41–2.17). An Italian study by Simonelli et al. (2017) [63] also established a relationship between the occurrence of endometriosis and exposure to BPA. Cobellis et al. (2009) [64] observed a detectable level of BPA in every woman with endometriosis but no detectable level in the control group.

In three studies, no relationship was found between BPA exposure and endometriosis [42,48,66].

In conclusion, the majority of the studies showed an association between BPA exposure and endometriosis, with only three studies observing no association.

### 3.3. Exposure to Parabens and Endometriosis

Parabens are commonly used as preservatives in food, cosmetic, and pharmaceutical products. These compounds are easily absorbed by the human body. Industrially, parabens are produced by esterification of PHBA (4-hydoxybenzoic acid) with appropriate alcohol in the presence of a catalyst. High efficiency and ease of reaction have contributed to the popularity of using parabens as a preservative [67]. Parabens may disrupt the hormonal balance by acting on enzymes responsible for the synthesis of estrogens or by modifying them to a free, unconjugated form [68]. In addition, parabens can also lead to hormonal disruptions in men. Exposure to parabens can result in androgen antagonistic activity, inhibition of sulfotransferase enzymes, and genotoxic activity [69]. Parabens are associated with increased levels of estradiol in healthy premenopausal women which can lead to irregular menstrual cycles. However, no relationship was observed between the concentration of paraben metabolites and the occurrence of polycystic ovary syndrome (PCOS) [70]. Parabens are components of feminine hygiene products, which may additionally result in increased exposure to this group of chemical compounds [71].

Only one study investigated the relationship between the development of endometriosis and exposure to parabens [72] (Table 3). Peinado et al. (2021) conducted a case-control study among women aged 20 to 54 years from the EndEA (Endometriosis y Exposicion Ambiental) study, involving two hospitals in Spain. A concentration of parabens was detected in urine samples. The women completed a questionnaire about lifestyle and the cosmetic products used. Endometriosis was confirmed by laparoscopy. A significant relationship was identified between the occurrence of endometriosis and the concentration of MeP (methylparaben) (OR: 5.63, *p* < 0.001). For the remaining examined parabens—EtP (ethylparaben), PrP (propylparaben), and BuP (buthylparaben)—no association was found.

As this was the first study to examine the relationship between exposure to parabens and endometriosis, it is difficult to draw conclusions. More studies should be performed in this direction to establish recommendations.

### 3.4. Exposure to Benzophenones and Endometriosis

Benzophenones (BP) are filters for ultraviolet light. BP absorbs mainly UV-B light. Due to their properties, they are used in the production of creams and products to protect human skin against the harmful effects of ultraviolet radiation [73]. As benzophenones are frequently used, many studies examined the relationship between exposure to benzophenones and reproductive and gynecological disorders. Studies found a correlation between exposure to benzophenones and fetal growth [74]. Fetal exposure to benzophenones may cause delayed growth, and the effects of exposure are more pronounced in female fetuses [74]. It was found that BP may affect reproductive function by interfering with estrogen receptors [75].

In two studies, the link between exposure to benzophenones and the occurrence of endometriosis was analyzed [72,76] (Table 4). In a case-control study performed by Peinado et al. (2021) [72] among women aged 20 to 54 years from the EndEA (Endometriosis y Exposicion Ambiental) study involving two hospitals in Spain, the concentrations of benzophenone-1 (BP-1), benzo-phenone-3(BP-3), and 4-hydroxibenzophenone (4-OH-BP) were determined in urine samples. There was a significant correlation between BP-1 (OR:5.12, *p* = 0.011) and BP-3 (OR: 4.98, *p* = 0.008) and the occurrence of endometriosis. A matched cohort study by Kunisue et al. (2012) [76] was performed among women aged 18–54 years participating in the ENDO project (Endometriosis, Natural History, Diagnosis, and Outcomes). The women were divided into two groups; women who had undergone laparoscopy or laparotomy (operative cohort), and women who were diagnosed with magnetic resonance imaging (population cohort). The research showed no relationship between the concentrations of 2-hydroxy-4-methoxybenzophenone (2OH-4MeO-BP), 2,4-dihydroxybenzophenone (2,4OH-BP), and 4-hydroxybenzophenone (4OH-BP) and the occurrence of endometriosis. As only two studies were performed on the effect of exposure to benzophenones on endometriosis, it is difficult to provide a conclusion.

### 3.5. Exposure to Non-Persistent Pesticides and Endometriosis

Organophosphorus (OP) and pyrethroid (PYR) pesticides are non-persistent endocrine disruptors [77,78]. OP is widely used in agriculture and horticulture to control plant pests. Organophosphorus pesticides exhibit toxicological effects by inhibiting the enzyme acetylcholinesterase [79]; moreover, OP can cause chronic neuropsychiatric disorders [80]. The relationship between exposure to pesticides and fetal death due to congenital abnormalities was investigated, revealing that exposure to pesticides between 3 and 8 weeks of pregnancy could lead to the assumed hypothesis [81]. It has been shown that the use of sprayers with OP as a component may have a negative impact on the quality of sperm in men [82]. Furthermore, organophosphorus pesticides could impair the functioning of the sexual endocrine system [83]. Pyrethroid pesticides are also used in agriculture and horticulture as insecticides. It is suspected that some may be carcinogenic, and it is also assumed that they may have a negative effect on the endocrine system [84]. A study was conducted showing that PYR exposure may have an effect on birth weight [85]. The effect of pyrethroids on the estrogenic and anti-progestogenic pathways was investigated, concluding that some pyrethroids may contribute to reproductive dysfunction [86].

Only one study examined the effect of non-persistent pesticides (organophosphate and synthetic pyrethroids) on the risk of developing endometriosis [87] (Table 5). The study by Li et al. (2020) [87] was carried out using a matched cohort study design among women aged 18–54 years participating in the ENDO project (Endometriosis, Natural History, Diagnosis, and Outcomes). The metabolites of the non-persistent pesticides were determined in urine samples (IMPY- 2-isopropyl-4-methyl-6-hydroxypyrimidine, MDA- malathion dicarboxylic acid, PNP-*para*-nitrophenol, TCPY-3,5,6-trichloro-2-pyridinol, 2,4-D—2,4-dichlorophenoxyacetic acid, 2,4,5-T—2,4,5-trichlorophenoxyacetic acid, 3-PBA—3-phenoxybenzoic acid, 4F-3PBA- 4-fluoro-3-phenoxybenzoic acid, *trans/cis*-DCCA-*trans/cis*-3-(2,2-dichlorovinyl)-2,2-dimethyl-cyclopropane-1-carboxylic acid, *cis*-DBCA-*cis*-3-(2,2-dibromovinyl)-2,2-dimethyl-cyclopropane-1-carboxylic acid). The women were divided into two groups, women who had undergone laparoscopy or laparotomy (operative cohort), and women who were diagnosed by magnetic resonance imaging (population cohort). The study found a significant association between endometriosis and urinary concentration of diazinon (the parent compound of IMPY) OR and chlorpyrifos and chlorpyrifos-methyl (parent compounds of TCPY) IMPY, (OR: 1.89; 95%, CI: 1.12–3.20) and TCPY, (OR: 1.65; 95% CI: 1.02–2.69).

**Table 1:** Exposure to phthalate and endometriosis.

Study	Study Design	Samples Measured	Concentration of Endocrine Disrupting Chemicals (EDC)		Study Population	RESULTS
Upson et al. (2013) [41] United States, Seattle, research was conducted among of a large healthcare system in the U.S. Pacific Northwest	population-based case-control	urinary concentration of MEHP, MEHHP, MEOHP, MECPP, MBzP, MEP, MiBP, MnBP LOQ = 0,2 ng/mL	Median (IQR): Cases: MEHP = 2.2 ng/mL (0.6–4.6) MEHHP = 14.8 ng/mL (5.3–31.0) MEOHP = 8.1 ng/mL (3.5–18.0) MECPP = 14.4 ng/mL (5.9–32.5) MBzP = 4.5 ng/mL (2.2–9.9) MEP = 61.9 ng/mL (23.5–155.9) MiBP = 1.3 ng/mL (0.6–2.7) MnBP = 9.8 ng/mL (5.0–20.9)	Median (IQR): Controls: MEHP = 3.4 ng/mL (1.0–11.1) MEHHP = 18.8 ng/mL (6.3–56.5) MEOHP = 10.8 ng/mL (3.5–29.1) MECPP = 18.0 ng/mL (5.8–51.9) MBzP = 5.0 ng/mL (2.0–11.5) MEP = 43.9 ng/mL (16.8–144.4) MiBP = 1.5 ng/mL (0.7–3.1) MnBP = 10.0 ng/mL (4.9–23.5)	287 reproductive-age women 92- cases 195- controls Women with endometriosis age range 18–49 years Women were recruited by in-person interviews covering a range of topics, including reproductive history and contraceptive use as well as medical and family history and lifestyle behaviors. The case group was surgically-confirmed cases and population-based controls. (1996–2001)	A strong inverse association between urinary MEHP concentration and endometriosis, when comparing the fourth and first MEHP quartiles (OR 0.3, 95% CI: 0.1–0.7). No statistically significant association between urinary concentrations of other DEHP metabolites MEHHP, MEOHP, and ∑DEHP and endometriosis, increased not statistically significant of endometriosis and urinary concentrations of MBzP and MEP was found.
Fernandez et al. (2019) [42] Brazil, Diagnosis of endometriosis was performed at the Endometriosis Center of Hospital School of the Federal University of Minas Gerais.	case-control study	urinary concentration of MMP, MiBP, MBP, MCHP, MiNP, MOP, MBzP, MEHP	Median: Cases: MMP = 57.6 µg/g MiBP = 129.8 µg/g MBP = 64.2 µg/g MCHP = 7.0 µg/g MEHP = 22.4 µg/g MiNP = 21.8 µg/g MOP = 670 µg/g MBzP = 23.8 µg/g	Median: Controls: MMP = 45.3 µg/g MiBP = 181.8 µg/g MBP = 72.4 µg/g MCHP = 4.6 µg/g MEHP = 21.2 µg/g MiNP = 14.7 µg/g MOP <LOQ MBzP < LOQ	52 women 30—cases 22- controls Women with endometriosis age range 18–45 years Participants signed a free informed consent form and filled out a questionnaire, with questions related to food habits, gynecological history, medicine intake as well as personal information. Criteria were confirmation of endometriosis by video laparoscopy surgery with histological diagnosis and the absence of the disease, respectively.	The phthalate metabolites that had the highest concentrations, were MOP and MiBP, in which the values of 670 µg/g and 560 µg/g, respectively. The relationship between endometriosis and all grouped metabolites was not statistically significant with *p* = 0.225.
Reddy et al. [43] (2006) India, The women were recruited at two centers: Bhagwan Mahavir Medical Hospital and Research Centre and Maternal Health and Reproductive Institute of Reproductive Medicine, Hyderabad, which receives cases from all over the region of Andhra Pradesh	case-control study	Serum cocncentration of DnBP, BBP, DnOP, DEHP	Arithmetic mean: Cases: DnBP = 0.44 µg/mL BBP = 0.66 µg/mL DnOP = 3.32 µg/mL DEHP = 2.44 µg/ml	Arithmetic mean: Controls I: DnBP = 0.08 µg/mL BBP = 0.12 µg/mL DnOP = 0 µg/mL DEHP = 0.5 µg/mL Controls II DnBP = 0.15 µg/mL BBP = 0.11 µg/mL DnOP = 0 µg/mL DEHP = 0.45 µg/mL	108 reproductive-age women. 49—cases 38—controls I 21—controls II Average age was 26.6 years in endometriosis cases, 27.4 years in control group I 27.1 years in control group II. Women completed a questionnaire to obtain information on the general obstetric and gynecological details including age at menarche, length of the menstrual cycle, associated symptoms, duration and amount of blood loss, duration of infertility, and socio-demographic details like age, body mass index (BMI) and limited information on diet were used for this study. The case group included 49 women who were diagnosed with endometriosis by laparoscopy. Group I comprised 38 women who attended the same hospital for other gynecological pathology (e.g., fibroids, tubal defects, polycystic ovaries, idiopathic infertility, and pelvic inflammatory disease) but were laparoscopically without endometriosis. Control group II comprised 21 women who attended the same hospital for laparoscopic tubal sterilization, with proven fertility and no evidence of endometriosis or other gynecological disorders.	The phthalate esters were observed in all samples for women with endometriosis. Besides correlation PEs with endometriosis was strong and statistically significant at *p* < 0.05 for all compounds DnBP: *p* < 0.0001; BBP: *p* < 0.0001; DnOP: *p* < 0.0001 DEHP: *p* < 0.0014
Cobellis et al. (2003) [44] Italy No information about the centers of treatment.	case-control study	Serum concentrations or peritoneal fluid of DEHP and MEHP	Serum concentrations Cases: Median DEHP = 0.57 µg/mL MEHP = 0.38 µg/mL Peritoneal fluid Cases: Median DEHP = 0.46 µg/mL MEHP = 0.37 µg/mL	Controls: Median DEHP = 0.18 µg/mL MEHP = 0.58 µg/mL	79 women 55—cases 24—controls Women with endometriosis age range 22–45 years. Women aged range 18–48 years without known infertility or reproductive diseases served in the controls. Diagnosis was confirmed by histological examination of the endometriotic lesions. Exclusion criteria were medical treatment for endometriosis or ovarian cyst before the surgery, and any surgical procedure in the previous 12 months.	Endometriotic women showed significantly higher plasma DEHP concentrations than controls. No significant differences in either the DEHP/MEHP plasma concentrations (*p* > 0.31) or DEHP/MEHP peritoneal fluid concentrations (*p* > 0.66) were observed in the endometriotic patients as a function of the disease stage at the time of diagnosis.
Huang et al. (2010) [45] Taiwan Women with endometriosis came from the Department of Obstetrics and Gynecology at a medical center in Taiwan.	Case-control study	Urinary concentration of MMP, MEP, MnBP, MBzP, 5oxo-MEHP, 5OH-MEHP, MEHP, ΣMEHP	Median Cases: MMP = 37.8 ng/mL/52.4 µg/g creatinine MEP = 31.6 ng/mL/58.0 µg/g creatinine MnBP = 60.2 ng/mL/94.1 µg/g creatinine MBzP = 5.6 ng/mL/12.2 µg/g creatinine 5oxo-MEHP = 12.1 ng/mL/19.0 µg/g creatinine 5OH-MEHP = 13.6 ng/mL/16.7 µg/g creatinine MEHP = 2.7 ng/mL 4.2 µg/g creatinine ΣMEHP = 28.7 ng/mL/42.4 µg/g creatinine	Median Controls: MMP = 28.1 ng/mL/32.1 µg/g creatinine MEP = 37.2 ng/mL/71.4 µg/g creatinine MnBP = 35.4 ng/mL/58.0 µg/g creatinine MBzP = 5.9 ng/mL/8.9 µg/g creatinine 5oxo-MEHP = 9.2 ng/mL/7.8 µg/g creatinine 5OH-MEHP = 5.7 ng/mL/9.9 µg/g creatinine MEHP = 0.8 ng/mL/3.4 µg/g creatinine ΣMEHP = 28.5 ng/mL/18.9 µg/g creatinine	57 women 28 with endometriosis 29—control group Women with endometriosis were recruited who underwent laparotomy and had pathologic confirmation of endometriosis. Controls were patients without any gynecologic conditions. (2005–2007)	In cases versus controls, an increased level of urinary mono-n-butyl phthalate (94.1 versus 58.0 µg/g creatinine, OR 3.46, Cl: 1.16–10.3, *p* < 0.05) was observed
Weuve et al. (2010) [46] United States Massachusetts The women were recruited from National Health and Nutrition Examination Survey (NHANES)	cross-sectional study	Urinary concentration of MEHP, MBP, MEP, MBzP, MEHHP, MEOHP	Geoetric mean (SD) Cases: MBP = 28.9 (4.1) ng/mg MEP = 207.0 (27.5) ng/mg MEHP = 2.5 (0.4) ng/mg MBzP = 14.4 (2.5) ng/mg MEHHP = 16.5 (2.8) ng/mg MEOHP = 11.5 (1.9) ng/mg	Geoetric mean (SD) Controls: MBP = 25.5 (1.0) ng/mg MEP = 219.9 (14.1) ng/mg MEHP = 3.4 (0.1) ng/mg MBzP = 14.1 (0.6) ng/mg MEHHP = 19.7 (1.4) ng/mg MEOHP = 13.5 (1.0) ng/mg	1227 women 87 with endometriosis 1020 control group Women was recruited 20–54 years of age. Sociodemographic information and medical histories of the survey participants and their families were collected during the household interviews Women were classified based on the history of endometriosis according to their response to the question “Has a doctor or other health professional ever told you that you had endometriosis?” (1999–2004)	No statistically significant association between assessed metabolites and endometriosis was found.
Nair et al. (2018) [47] Pakistan/ Australia This study was financially supported by the University of Health Sciences, Lahore, Pakistan.	case-control study	Serum concentration of DEHP	Arithmetic mean: Cases: DEHP = 65.29 (ng/mL) Arithmetic mean in all the four stages of endometriosis: DEHP stage I = 60.4 ng/mL DEHP stage II = 58.68 ng/mL DEHP stage III = 66.25 ng/mL DEHP stage IV = 68.11 ng/mL	Arithmetic mean: Controls: Not detected	100 women 50 with endometriosis 50 without endometriosis Women with endometriosis age range 20–40 years. Only cases were included who were infertile and had been declared positive for endometriosis after laparoscopy by expert surgeons, according to the criteria set by the American Society for Reproductive Medicine (ASRM, 2012). ASRM guidelines classify endometriosis into four stages: I—minimal, II—mild, III—moderate, and IV—severe. For controls, due to ethical considerations, laparoscopy was not done.	DEHP exposure was associated with advanced stages III and IV of endometriosis.
Louis et al. (2013) [48] United States, Utah The women were recruited in 14 participating clinical centers in the Salt Lake City, Utah, and San Francisco, California geographic areas.	matched cohort design	Urinary concentration of phthalate metabolites: MMP, MEP, MCPP, MBP, MiBP, MECPP, MCMHP, MEHHP, MEOHP, MCHP, MBzP, MEHP	Geometric mean Operative Cohort: Endometriosis: MMP = 2.12 ng/mL MEP = 107.2 ng/mL MCPP = 2.71 ng/mL MBP = 12.07 ng/mL MiBP = 7.28 ng/mL MECPP = 24.68 ng/mL MCMHP = 29.34 ng/mL MEHHP = 16.34 ng/mL MEOHP = 10.98 ng/mL MCHP = 0.03 ng/mL MBzP = 6.96 ng/mL MEHP = 4.75 ng/mL MOP = 0.06 ng/mL MNP = 0.16 ng/mL Without endometriosis: MMP = 2.35 ng/mL MEP = 109.6 ng/mL MCPP = 3.41 ng/mL MBP = 11.01 ng/mL MiBP = 6.82 ng/mL MECPP = 24.98 ng/mL MCMHP = 29.19 ng/mL MEHHP = 14.40 ng/mL MEOHP = 10.12 ng/mL MCHP = 0.04 ng/mL MBzP = 7.82 ng/mL MEHP = 4.12 ng/mL MOP = 0.06 ng/mL MNP = 0.16 ng/mL	Geometric mean Population Cohort Endometriosis: MMP = 3.67 ng/mL MEP = 152.0 ng/mL MCPP = 5.75 ng/mL MBP = 19.13 ng/mL MiBP = 13.32 ng/mL MECPP = 54.15 ng/mL MCMHP = 53.54 ng/mL MEHHP = 32.37 ng/mL MEOHP = 23.03 ng/mL MCHP = 0.04 ng/mL MBzP = 9.85 ng/mL MEHP = 8.32 ng/mL MOP = 0.06 ng/mL MNP = 0.22 ng/mL Without endometriosis: MMP = 2.71 ng/mL MEP = 138.2 ng/mL MCPP = 4.06 ng/mL MBP = 11.24 ng/mL MiBP = 7.59 ng/mL MECPP = 20.27 ng/mL MCMHP = 22.51 ng/mL MEHHP = 11.86 ng/mL MEOHP = 8.29 ng/mL MCHP = 0.03 ng/mL MBzP = 6.46 ng/mL MEHP = 3.07 ng/mL MOP = 0.05 ng/mL MNP = 0.16 ng/mL	626 women 495 operative cohort 131 population cohort Women was recruited 18–44 years of age. In-person standardized interviews were conducted with women prior to surgery or MRI followed by an anthropometric assessment Operative cohort was undergoing laparoscopy/laparotomy. Endometriosis was confirmed in 190 women, and 283 were without endometriosis. The population cohort received standardized pelvic magnetic resonance imaging (MRI) for the assessment of endometriosis. 127 women were confirmed with endometriosis in the population cohort. (2007–2009)	A significant association was observed between MBP, MCMHP, MECPP, MEHP, MEHHP, and MEOHP and endometriosis in the population cohort. No significant association between urinary concentration phthalates occurrence and endometriosis in the operative cohort.
Itoh et al. (2009) [49] Japan The women were recruited from the Department of Obstetrics and Gynecology of the Jikei University School of Medicine for the treatment of infertility.	case-control study	Urinary concentration of MEP, MnBP, MBzP, MEHP, MEHHP, MEOHP	Cases (II-IV stages): Median unadjusted for creatinine (creatinine-adjusted) MEP = 39.6 µg/L (18.9 µg/g creatinine) MnBP = 87.2 µg/L (47.6 µg/g creatinine) MBzP = 3.9 µg/L (2.1 µg/g creatinine) MEHP = 10.2 µg/L (4.9 µg/g creatinine) MEHHP = 39.6 µg/L (19.2 µg/g creatinine) MEOHP = 39.5 µg/L (19.1 µg/g creatinine)	Controls (0–1 stages): Median unadjusted for creatinine (creatinine-adjusted) MEP = 21.4 µg/L (11.2 µg/g creatinine) MnBP = 84.3 µg/L (43.3 µg/g creatinine) MBzP = 3.2 µg/L (1.8 µg/g creatinine) MEHP = 8.3 µg/L (4.2 µg/g creatinine) MEHHP = 32.2 µg/L (17.3 µg/g creatinine) MEOHP = 32.1 µg/L (16.3 µg/g creatinine)	137 women Women were recruited 20–45 years of age. Participants were interviewed before laparoscopic examination by a single trained interviewer using a structured questionnaire to collect information on demographic factors, age, height, weight, personal and family medical, reproductive and menstrual history, oral contraceptive use, food, and alcohol consumption, and smoking history. The severity of endometriosis was diagnosed using laparoscopy. Cases of endometriosis have been classified into five stages based on the American Fertility Society classification and then categorized into: 80 controls (stage 0 or I) 57 cases (stages II-IV). (I-minimal, II-mild, III-moderate, and IV-severe)	No significant association between endometriosis and urinary concentration of phthalate.
Kim et al. (2015) [50] Korea The women were recruited from the department of Obstetrics & Gynecology in Asan Medical Center, Seoul, Korea.	case-control study	Urinary concentration of: MEHHP, MEOHP, MnBP, MECPP	Arithmetic mean ± SE Cases: MEHHP = 18.2 ± 1.7 µg/g creatinine MEOHP = 13.4 ± 1.1 µg/g creatinine MnBP = 41.7 ± 6.2 µg/g creatinine MBzP = 5.8 ± 1.0 µg/g creatinine MECPP = 23.8 ± 1.9 µg/g creatinine	Arithmetic mean ± SE Controls: MEHHP = 12.9 ± 1.4 µg/g creatinine MEOHP = 10.3 ± 0.9 µg/g creatinine MnBP = 32.4 ± 3.1 µg/g creatinine MBzP = 7.3 ± 1.9 µg/g creatinine MECPP = 19.0 ± 1.7 µg/g creatinine	88 women 55—cases 33—controls The mean age in the endometriosis group was 29.9 years and in the control group was 32.6 years. Any women with a history of occupational exposure to reproductive toxicants, smoking, alcohol, and other addictions were excluded from this study. Th endometriosis group had undergone pelviscopic surgery, exploratory laparotomy, or transabdominal hysterectomy. (2012–2013)	Significant association between urinary concentrations of phthalates and endometriosis: MEHHP OR = 2.52; *p* = 0.041 MEOHP OR = 2.89; *p* = 0.043 MECPP OR = 2.57; *p* = 0.071
Kim et al. (2011) [51] Korea The women were recruited from the department of Obstetrics and Gynecology in Asan Medical Center, Seoul, Korea	case-control study	Serum concentration of MEHP, DEHP	Arithmetic mean ± SE Cases: MEHP = 17.4 ± 1.5 ng/mL DEHP = 179.7 ± 32.5 ng/mL	Arithmetic mean ± SE Controls: MEHP = 12.4 ± 1.1 ng/mL DEHP = 92.5 ± 31.1 ng/mL	266 women 97—cases 169—controls All of the subjects recruited in this study were from urban areas, without a history of occupational exposure to reproductive toxicants, smoking, alcohol, and other addictions. This study comprised a total of 266 patients who had undergone pelviscopic surgery, exploratory laparotomy, myomectomy, or transabdominal hysterectomy. The endometriosis group had surgical and histologic evidence of advanced-stage endometriosis. (January 2009 and September 2009.	Plasma levels of MEHP were significantly higher in those with advanced-stage of endometriosis (levels of phthalate esters might be quite different between the patients with stage I–II and III–IV endometriosis). MEHP OR = 1.020(1.003–1.038)/*p* = 0.020 No significant association between DEHP and endometriosis. DEHP OR= 1.001 (1.000–1.002)/*p* = 0.161
Zhang et al. (2021) [52] China The women were recruited from the National Health and Nutrition Examination Survey (NHANES)	cross-sectional study	Urinary concentration of: MBP, MCHP, MEP, MEHP, MNP, MOP, MBzP, MNM, MCPP, MEHHP, MEOHP, MIBP	Geometric mean for the whole population: MBP = 20.89 ng/mL MCHP = 0.45 ng/mL MEP = 160.27 ng/mL MEHP = 3.55 ng/mL MNP = 1.19 ng/mL MOP = 1.32 ng/mL MBzP = 8.80 ng/mL MNM = 1.65 ng/mL MCPP = 2.16 ng/mL MEHHP = 20.06 ng/mL MEOHP = 14.03 ng/mL MIBP = 4.13 ng/mL		1204 women 77—cases 1127—controls Women were recruited from 20–54 years of age, information about endometriosis was based on a questionnaire. (2001–2006)	No association between examined phthalates metabolites and endometriosis.

Abbreviations: LOQ—limit of quantitation, IQR—interquartile range, OR—odds ratio, SD—standard deviation, SE—standard error, MEHP—mono-(2-ethyl)-hexyl phthalate, MEHHP—mono-(2-ethyl-5-hydroxyhexyl) phthalate, MEOHP—mono-(2-ethyl-5-oxohexyl) phthalate, MECPP—mono-(2-ethyl-5-carboxypentyl) phthalate, MBzP—mono-benzyl phthalate, MEP—monoethyl phthalate, MiBP—mono-iso-butyl phthalate, MnBP—mono-benzyl phthalate, MMP—mono-methyl phthalate, MBP—mono-butyl phthalate, MCHP—mono-cyclohexyl phthalate, MiNP—mono-isononyl phthalate, DnBP—di-n-butyl phthalate, BBP—butyl benzyl phthalate, DnOP—di-n-octyl phthalate, DEHP—diethylhexyl phthalate, 5oxo-MEHP—mono-(2-ethyl-5-oxo-hexyl) phthalate, 5OH-MEHP- mono-(2-ethyl 5-hydroxyhexyl) phthalate, MCPP—mono (3-carboxypropyl) phthalate, MCMHP—mono-[(2-carboxymethyl) hexyl] phthalate, MNP—mono-isononyl phthalate, MOP—mono-octyl phthalate, MNM—mono-n-methyl phthalate, MIBP—mono-isobutyl phthalate.

**Table 2:** Exposure to Bisphenol-A and endometriosis.

Study	Study Design	Samples Measured	Concentration of Endocrine Disrupting Chemicals (EDC)		Study Population	Results
Fernandez et al. (2019) [42] Brazil Diagnosis of endometriosis was performed at the Endometriosis Center of Hospital School of the Federal University of Minas Gerais.	case-control study	Urinary concentration of BPA	Median Cases: BPA = 8.9 µg/g	Median: Controls: BPA = 8.8 µg/g	52 women 30—cases 22—controls Women with endometriosis age range 18–45 years Participants signed a free informed consent form and filled out a questionnaire, with questions related to food habits, gynecological history, medicine intake as well as personal information. Criteria were confirmation of endometriosis by video laparoscopy surgery with histological diagnosis and the absence of the disease, respectively.	No association between BPA in the urine and endometriosis.
Itoh et al. (2007) [66] Japan Women were recruited from the Department of Obstetrics and Gynecology of the Jikei University School of Medicine for the treatment of infertility	cross-sectional study	Urinary concentration of BPA	Median BPA = 1.57 µg/L (0.8 µg/g creatine)		140 women The women with endometriosis age range 20–45 years. Interviews using a questionnaire to collect information on demographic factors, age, height, weight, personal and family medical, reproductive and menstrual histories, oral contraceptive use, food, and alcohol consumption frequencies, and smoking history. The endometriosis was diagnosed using laparoscopy and then classified into five stages on the basis of the revised American Fertility Society classification: stage 0 (*n* = 60), I (*n* = 21), II (*n* = 10), III (*n* = 24), and IV (*n* = 25).	No association between urinary BPA concentration and endometriosis, *p* = 0.24
Upson et al. (2014) [60] United States The women were recruited from Group Health (GH), a large integrated healthcare system in the US Pacific Northwest	case-control study	Urinary concentration of BPA	Percentage of cases with different quartiles of exposure to BPA Cases: Quartiles ≤0.364 = 21.7% >0.364–0.863 = 23.1% >0.863–2.01 = 28.0% >2.01 = 27.3% Ovarian endometriosis Cases: ≤0.364 = 29.3% >0.364–0.863 = 14.7% >0.863–2.01 = 24.0% >2.01 = 32.0% Non-ovarian pelvic endometriosis ≤0.364 = 13.2% >0.364–0.863 = 32.4% >0.863–2.01 = 32.4% >2.01 = 22.1%	Percentage of cases with different quartiles of exposure to BPA Controls: Quartiles ≤0.364 = 25.1% >0.364–0.863 = 25.1% >0.863–2.01 = 25.1% >2.01 = 24.7%	430 women 143—cases 287—controls The women with endometriosis age range 18–49 years. In-person interview that occurred after case diagnosis, eliciting detailed reproductive, contraceptive, medical, and family history as well as lifestyle behavior information. Cases were women first diagnosed with endometriosis, with medical record confirmation of direct surgical visualization of endometriosis, and also cases had pathology-confirmed endometriosis. Controls were women identified from computerized GH enrollment databases, without a current or prior diagnosis of endometriosis. (1996–2001)	A statistically significant association between total urinary BPA concentrations and endometriosis overall. Statistically significant positive associations when evaluating total urinary BPA concentrations in relation to non-ovarian pelvic endometriosis (second versus lowest quartile: OR 3.0; 95% CI: 1.2, 7.3; third versus lowest quartile: OR 3.0; 95% CI: 1.1, 7.6), but not in relation to ovarian endometriosis
Peinado et al. (2020) [61] Spain The women were recruited from the Surgery and the Gynecology and Obstetrics Units of the San Cecilio and Virgen de las Nieves University Hospitals in Granada, southern Spain	case-control study	Urinary concentrations of BPA	Geometric mean ± SD Cases: BPA = 5.5 (1.1) ng/mL	Geometric mean ± SD Controls: BPA = 3.0 (1.2) ng/mL	124 women 35—case 89—control The women with endometriosis age range 20–54 years. Women underwent clinical and anthropometrical examination, calculating their BMI from their height and weight. Surgical and clinical questionnaires were used to gather sociodemographic, lifestyle, clinical, and surgical data, including residence, educational level, occupational status, current smoking, parity, and the average level of menstrual bleeding. Cases were women with endometriosis diagnosed by laparotomy or laparoscopic surgery and histological confirmation, while controls were women undergoing abdominal surgery for non-malign diseases (including acute appendicitis, biliary disease, hiatus hernia, ovarian torsion, corpus luteum, uterine fibroids, and cystadenomas, among others) in whom the absence of endometrial lesions was visually and histologically confirmed. (2018–2019)	Association between BPA concentrations and endometriosis OR = 1.5; *p* < 0.05
Rashidi et al. (2017) [62] Iran This case-control study was approved by the Institutional Review Board of Tehran University of Medical Sciences. The samples were analyzed at Pharmaceutical Science Research Center of Tehran University of Medical Sciences	case-control study	Urinary concentrations of BPA	Geometric mean ± SD Cases: BPA = 5.53 ± 3.46 ng/mL	Geometric mean ± SD Controls: BPA = 1.42 ± 1.56 ng/mL	100 women 50—case 50—control The women with endometriosis age range 22–45 years. Women filled out a questionnaire about lifestyle. Women with endometriosis were candidates for operative laparoscopy and ovarian cystectomy as cases. Control group was women who had not any evidence of endometrioma in clinical and ultrasound evaluation and came to the same clinic.	Association between the BPA urinary concentrations among women with endometrioma were statistically higher compared with the control group. Crude OR 1.75; *p* < 0.001
Louis et al. (2013) [48] United States Utah The women were recruited in 14 participating clinical centers in the Salt Lake City, Utah, and San Francisco, California geographic areas.	matched cohort design	Urinary concentration of BPA	Geometric mean Operative Cohort: Endometriosis: BPA = 1.45 mg/dL Without endometriosis: BPA = 1.66 mg/dL	Geometric mean Population Cohort: Endometriosis: BPA = 4.19 mg/dL Without endometriosis: BPA = 1.65 mg/dL	626 women 495 operative cohort 131 population cohort Women were recruited 18–44 years of age. In-person standardized interviews were conducted with women prior to surgery or MRI followed by an anthropometric assessment Operative cohort was undergoing laparoscopy/laparotomy. Endometriosis was confirmed in 190 women, and 283 were without endometriosis. The population cohort had standardized pelvic magnetic resonance imaging (MRI) for the assessment of endometriosis. 127 women were confirmed with endometriosis in the population cohort. (2007–2009)	No association between BPA concentration and endometriosis in the operative cohort and population cohort.
Simonelli et al. (2016) [63] Italy Women with endometriosis and endometriosis-free subjects were referred to the outpatient infertility clinic at the Second University of Naples.	case-control study	Urinary concentration of BPA	Arithmetic mean Endometriosis: BPA = 5.31 ± 3.36 pg/µl	Arithmetic mean Control: BPA = 1.64 ± 0.49 pg/µl	128 women 68—case 60—control The age of the group was not provided in the study, the author included age ranges (x < 30; 30 ≤ x ≥ 35; 35 ≤ x ≥ 40; x ≥ 40). A questionnaire investigating the occupational context, living environment, and habits was administered to patients suffering from endometriosis and endometriosis-free subjects (control group). All women with a regular menstrual cycle undergoing laparoscopy because of infertility, chronic pelvic pain, or sonographic diagnosis of ovarian endometriosis were recruited. Laparoscopy was performed in the proliferative phase, evidenced by hormone and ultrasonography (USG) analyses. Sixty-eight women were diagnosed with a histologically confirmed diagnosis of endometriosis; 60 women were control group.	A statistically significant difference between patients and controls, showing an association between BPA exposure and endometriosis.
Cobellis et al. (2009) [64] Italy The study was approved by the institutional review board of the Second University of Naples; informed consent was obtained from the participants. A group of fertile women referred to the Department of Gynaecology, Obstetrics, and Reproductive Medicine of the same university was enrolled.	case-control study	Serum concentration of BPA	Arithmetic mean ± SD Cases: BPA = 2.91 ± 1.74 ng/mL	Arithmetic mean: Controls: Not detected	69 women 58—cases 11—controls The women with endometriosis age range 18–44 years. No information about lifestyle from a questionnaire. The patients were submitted to diagnostic or operative laparoscopy for the evidence of ovarian cysts or to investigate chronic pelvic pain and dysmenorrhea The endometriosis diagnosis was confirmed by histological examination of the endometriotic lesions, and patients were classified according to the revised American Fertility Society classification of endometriosis.	BPA has not been found in any of the between from healthy women (control group). However, BPA has been observed in 30 women with endometriosis.
Wen et al. (2020) [65] China The women were recruited from the Obstetrics and Gynecology Department of Zhongnan Hospital, Wuhan University. A control group with another 100 cases of healthy women who sought help because of their husband’s infertility in the Reproductive Medicine Center or who had a routine physical examination in the Medical Examination Center of Zhongnan Hospital, Wuhan University, were also recruited as the control group.	case-control study	Urinary concentration of BPA	Median (IQR) Cases: BPA = 1.55 (0.85–1.95) µg/g	Median (IQR) Controls: BPA = 1.30 (0.74–1.99) µg/g	220 women 120—cases 100—controls Women were recruited 20–50 years of age. Women completed a questionnaire about lifestyle. Women undergoing laparoscopy were defined as EMs histopathologically. Patients with ovarian EMs who displayed peritoneal lesions were excluded from this study. Finally, 120 patients (including 73 cases of ovarian EMs, 47 cases of peritoneal EMs) were recruited as the case group. In this study, controls were women who met the following three conditions: aged from 20 to 50 years; without a current or prior diagnosis of EMs or infertility; and had no ovarian or other pelvic, abdominal masses suggested by B-ultrasound. All subjects were in the proliferative phase of the menstrual cycle determined by their menstrual history and were free of any hormone treatment for > 3 months prior to sample collection. Other gynecologic diseases such as adenomyosis, hysteromyoma, endometrial polyp, and polycystic ovary syndrome were also excluded. (2017–2018)	Association between the BPA urinary concentrations among women with endometrioma were higher compared with the control group. The risk of peritoneal EMs increased approximately tenfold when creatinine-adjusted urinary BPA concentration was 2 μg/g.

Abbreviations: BPA—bisphenol A, OR—odds ratio, SD—standard deviation.

**Table 3:** Exposure to parabens and endometriosis.

Study	Study Design	Samples Measured	Concentration of Endocrine Disrupting Chemicals (EDC)		Study Population	Results
Peinado et al. (2020) [72] Spain The women with endometriosis was part of the hospital-based case-control EndEA study (Endometriosis y Exposicion Ambiental), in two public hospitals (‘San Cecilio’ and ‘Virgen de las Nieves’) in Granada, Southern Spain	case-control study	Urinary concentration of MeP, EtP, PrP, BuP	Arithmetic mean ± SD Cases: MeP = 210.98 ± 512.79 ng/mL EtP = 35.18 ± 79.27 ng/mL PrP = 14.35 ± 30.22 ng/mL BuP = 1.15 ± 3.42 ng/mL	Arithmetic mean ± SD Controls: MeP = 148.91 ± 272.94 ng/mL EtP = 37.12 ± 118.82 ng/mL PrP = 8.59 ± 18.87 ng/mL BuP = 1.40 ± 2.25 ng/mL	124 women 35—case 89—control The women with endometriosis age range 20–54 years. The women filled out a questionnaire about lifestyle and used cosmetics products. Endometriosis was confirmed (cases) or ruled out (controls) by laparoscopy, with a visual inspection of the pelvis and biopsy of suspected lesions (histological diagnosis) Inclusion criteria were: receipt of abdominal surgery (laparotomy or laparoscopy) and pathology report on the presence or absence of endometriosis. Further criteria for controls were: performance of laparotomy or laparoscopy in the same hospital as cases for non-malignant disease (e.g., acute appendicitis, biliary disease, hiatus hernia, ovarian torsion, corpus luteum, and cystadenomas, among others), no findings of endometriosis during the surgery, and no history of endometriosis. (2018–2019)	Significant association between endometriosis and urinary concentration of MeP (OR 5.63, *p* < 0.001). No association with others examined parabens and endometriosis.

Abbreviations: OR—odds ratio, SD—standard deviation, MeP—methylparaben, EtP—ethylparaben, PrP—propylparaben, BuP—buthylparaben.

**Table 4:** Exposure to benzophenones and endometriosis.

Study	Study Design	Samples Measured	Concentration of Endocrine Disrupting Chemicals (EDC)		Study Population	Results
Peinado et al. (2020) [72] Spain The women with endometriosis were, part of the hospital-based case-control EndEA study (Endometriosis y Exposicion Ambiental), in two public hospitals (‘San Cecilio’ and ‘Virgen de las Nieves’) in Granada, Southern Spain	case-control study	Urinary concentration of BP-1, BP-3, 4-OH-BP	Arithmetic mean ± SD Case: BP-1 = 3.37 ± 4.99 ng/mL BP-3 = 10.84 ± 32.61 ng/mL 4-OH-BP = 0.99 ± 1.01 ng/mL	Arithmetic mean ± SD Control: BP-1 = 22.73 ± 107.95 ng/mL BP-3 = 35.07 ± 115.36 ng/mL 4-OH-BP = 3.26 ± 19.61 ng/mL	124 women 35—case 89—control The women with endometriosis age range 20–54 years. The women filled out a questionnaire about lifestyle and used cosmetics products. Endometriosis was confirmed (cases) or ruled out (controls) by laparoscopy, with a visual inspection of the pelvis and biopsy of suspected lesions (histological diagnosis) Inclusion criteria were: receipt of abdominal surgery (laparotomy or laparoscopy), and pathology report on the presence or absence of endometriosis. Further criteria for controls were: performance of laparotomy or laparoscopy in the same hospital as cases for non-malignant disease (e.g., acute appendicitis, biliary disease, hiatus hernia, ovarian torsion, corpus luteum, and cystadenomas, among others), no findings of endometriosis during the surgery, and no history of endometriosis. (2018–2019)	Significant association between endometriosis and urinary concentration of BP-1 (OR = 5.12, *p* = 0.011) and BP-3 (OR = 4.98, *p* = 0.008). Others benzophenones have not been associated with endometriosis.
Kunisue et al. (2012) [76] United States Utah and California Urine samples were collected for the ENDO (Endometriosis, Natural history, Diagnosis, and Outcomes) from women who resided within 50 miles of two cities, Salt Lake City (Utah) and San Francisco (California)	matched cohort study	Urinary concentration of 2OH-4MeO-BP, 2,4OH-BP, 4OH-BP	Median for the study cohort sum 2OH-4MeO-BP = 6.1 ng/mL 2,4OH-BP = 6.1 ng/mL 4OH-BP = 0.36 ng/mL		600 women 473—operative cohort 127—population cohort The women with endometriosis age range 18–44 years. The women filled out a questionnaire about lifestyle The women underwent laparoscopy/laparotomy (operative cohort) or pelvic magnetic resonance imaging (population cohort). Endometriosis diagnoses were categorized into four stages: minimal, mild, moderate, and severe according to the Revised American Society for Reproductive Medicine’s classification. (2007–2009)	No association between urinary concentration and benzophenone-type UV Filters and endometriosis. Operative cohort: 2OH-4MeO-BP, OR = 1.08, 2,4OH-BP, OR = 1.19, 4OH-BP, OR = 0.97, Population cohort: 2OH-4MeO-BP, OR = 1.25, 2,4OH-BP, OR = 1.03, 4OH-BP, OR = 1.19,

Abbreviations: OR—odds ratio, SD—standard deviation, BP-1—benzophenone-1, BP-3—benzo-phenone-3, 4-OH-BP-4-hydroxibenzophenone, 2OH-4MeO-BP—2-hydroxy-4-methoxybenzophenone, 2,4OH-BP—2,4-dihydroxybenzophenone, 4OH-BP—4-hydroxybenzophenone.

**Table 5:** Exposure to nonpersistent pesticides and endometriosis.

Study	Study Design	Samples Measured	Concentration of Endocrine Disrupting Chemicals (EDC)	Study Population	Results
Li et al. (2020) [87] United States Utah and California Urine samples were collected for the ENDO (Endometriosis, Natural history, Diagnosis, and Outcomes) from women who resided within 50 miles of two cities, Salt Lake City (Utah) and San Francisco (California)	matched cohort study	Urinary concentration of IMPY, MDA, PNP, TCPY, 2,4-D, 2,4,5-T, 3-PBA, 4F-3PBA, trans-DCCA, cis-DCCA, cis-DBCA	Median for the study cohort sum IMPY = 2.70 ng/mL MDA = 0.217 ng/mL PNP = 0.637 ng/mL TCPY = 0.601 ng/mL 2,4-D = 0.249 ng/mL 2,4,5-T < LOD 3-PBA = 0.166 ng/mL 4F-3PBA = 0.008 ng/mL trans-DCCA = 0.055 ng/mL cis-DCCA = 0.091 ng/mL cis-DBCA < LOD	594 women 471—operative cohort 123—population cohort The women with endometriosis age range 18–44 years. The women filled out a questionnaire about lifestyle The women underwent laparoscopy/laparotomy (operative cohort) or pelvic magnetic resonance imaging (population cohort). Endometriosis diagnoses were categorized into four stages: minimal, mild, moderate, and severe, according to the Revised American Society for Reproductive Medicine’s classification. (2007–2009)	A significant association between endometriosis and urinary concentration of diazinon (the parent compound of IMPY) and chlorpyrifos and chlorpyrifos-methyl (parent compounds of TCPY) IMPY, OR = 1.89 TCPY, OR = 1.65

Abbreviations: OR—odds ratio, IMPY—2-Isopropyl-4-methyl-6-hydroxypyrimidine, MDA—malathion dicarboxylic acid, PNP—3,5,6-trichloro-2-pyridinol, TCPY—3,5,6-trichloro-2-pyridinol, 4F-3PBA—4-fluoro-3-phenoxybenzoic acid, 3-PBA—3-phenoxybenzoic acid, 2,4-D—2,4-dichlorophenoxyacetic acid, 2,4,5-T—2,4,5-trichlorophenoxyacetic acid, trans/cis-DCCA—trans/cis-3-(2,2-dichlorovinyl)-2,2-dimethyl-cyclopropane-1-carboxylic acid, cis-DBCA—cis-3-(2,2-dibromovinyl)-2,2-dimethyl-cyclopropane-1-carboxylic acid.

## 4. Discussion

Most of the studies reviewed in this paper showed an association between exposure biomarkers and non-persistent EDCs, with endometriosis, involving at least one metabolite of these compounds (Table 6). Five of the reviewed studies showed an association between the concentration of BPA and endometriosis, while four observed no association.

In the case of phthalates exposure, the seven reviewed studies found an association between the concentration of at least one phthalate metabolite and endometriosis whereas most studies on single compounds indicated no significant association. Additionally, several studies included up to 10 metabolites, increasing the risk of random associations. Five studies found no association between phthalate concentration and endometriosis. Only one study was performed to assess the link between exposure to parabens and endometriosis, finding a significant relationship between the concentration of MeP and endometriosis. For the remaining examined parabens—EtP, PrP, and BuP—no association was found. Additionally, only one study assessed the effect of exposure to non-persistent pesticide exposure on endometriosis, observing a significant association between endometriosis and the urinary concentration of diazinon (the parent compound of IMPY) as well as chlorpyrifos and chlorpyrifos-methyl (parent compounds of TCPY).

Only two studies were performed on the effect of exposure to benzophenones on endometriosis. Their results were inconclusive, making it difficult to provide a conclusion on this effect. A comparison of the reviewed studies is presented in Table 6.

The inconsistencies in the results may have been due to many limitations of the presented studies, such as differences in the confounding factors used in the statistical models, study design, study population, biomarkers of exposure, biological fluids used for assessment, creatinine or specific gravity adjustment, time of exposure, and outcome assessment (diagnosis *versus* questionnaire data).

In most of the presented studies, a case-control study design was used, which is often used to identify factors that may contribute to a medical condition by comparing subjects who have that condition/disease (cases) with those who do not but are otherwise similar (controls). On the other hand, case-control studies also have some limitations, whereby associations measured may or may not represent causal relationships. It can be hard to establish if there is true temporality (i.e., if the exposure preceded the outcome; or vice versa). Furthermore, cases and controls may have different recollections of exposure, leading to a unique source of bias. The study populations were mostly recruited mostly from fertility and gynecology centers. Non-persistent endocrine-disrupting chemicals were analyzed in urine in most of the reviewed studies. Additionally, the authors did not state the number of analyzed urine samples collected from each patient. As non-persistent endocrine disruptors are metabolized in 24–48 h, a single urine sample may not reliably define exposure and its association with endometriosis. However, as people do not change their lifestyle very often, exposure is typically habitual. Thus, as reported by Meeker et al. (2005) [88], a single sample can adequately predict longer-term average exposure. The outcomes (endometriosis) in most presented studies were assessed by surgery or magnetic resonance imaging. However, in two studies, the diagnosis was based on questionnaire data. In most studies, similar confounding factors were used in the statistical models, e.g., age, smoking status, body mass, age at menarche, education level, and pregnancy status.

In the case of studies investigating phthalate exposure, the divergence of the results may have arisen from the various confounding factors identified in the studies, such as differences in creatinine adjustment, sample size, study design, phthalate metabolites assessed, and biological fluids in which the concentrations of phthalates were measured. In studies of the effect of BPA exposure on endometriosis, the inconsistent results may have been due to the differences in the selection of study groups. In benzophenone studies, the use of diverse biomarkers (different benzophenones) and various confounding factors may have affected the results.

## 5. Conclusions

In conclusion, despite the numerous limitations of the results, the reviewed studies suggest that exposure to non-persistent endocrine disruptors, especially in the case of bisphenol A and phthalates is associated with endometriosis. The results of the studies on parabens, benzophenones, and non-persistent insecticides were inconclusive.

The studies were mostly well-designed epidemiological studies, using biomarkers of exposure, where the outcome (endometriosis) was based on a confirmed diagnosis. Additionally, the statistical models were adjusted for potential confounding factors.

Due to the insufficient evidence, further epidemiological studies are needed to confirm these findings.

## Figures and Tables

**Table 6:** Exposure to non-persistent endocrine disrupting chemicals exposure and endometriosis.

Chemical Compound	Endometriosis
**Phthalates:** MEHP	+ Upson et al. (2013) [41]; Fernandez et al. (2019) [42]; Louis et al. (2013) [48]; Kim et al. (2011) [51] - Cobellis et al. (2003) [44]; Huang et al. (2010) [45]; Weuve et al. (2010) [46]; Itoh et al. (2009) [49]; Zhang et al. (2021) [52]
MEHHP	+ Louis et al. (2013) [48]; Kim et al. (2015) [50] - Upson et al. (2013) [41]; Weuve et al. (2010) [46]; Itoh et al. (2009) [49]; Zhang et al. (2021) [52]
MEOHP	+ Louis et al. (2013) [48]; Kim et al. (2015) [50] - Upson et al. (2013) [41]; Weuve et al. (2010) [46]; Itoh et al. (2009) [49]; Zhang et al. (2021) [52]
MECPP	+ Louis et al. (2013) [48]; Kim et al. (2015) [50] - Upson et al. (2013) [41]
MBzP	- Upson et al. (2013) [41]; Fernandez et al. (2019) [42]; Louis et al. (2013) [48]; Huang et al. (2010) [45]; Weuve et al. (2010) [46]; Itoh et al. (2009) [49]; Zhang et al. (2021) [52]
MEP	- Upson et al. (2013) [41]; Huang et al. (2010) [45]; Weuve et al. (2010) [46]; Louis et al. (2013) [48]; Itoh et al. (2009) [49]; Zhang et al. (2021) [52]
MiBP	- Upson et al. (2013) [41]; Fernandez et al. (2019) [42]; Louis et al. (2013) [48]; Zhang et al. (2021) [52]
MnBP	+ Huang et al. (2010) [45] - Upson et al. (2013) [41]; Itoh et al. (2009) [49]; Kim et al. (2015) [50]
MMP	- Fernandez et al. (2019) [42]; Louis et al. (2013) [48]; Huang et al. (2010) [45]
MBP	+ Louis et al. (2013) [48] - Fernandez et al. (2019) [42]; Weuve et al. (2010) [46]; Zhang et al. (2021) [52]
MCHP	- Fernandez et al. (2019) [42]; Louis et al. (2013) [48]; Zhang et al. (2021) [52]
MiNP	- Fernandez et al. (2019) [42]
DnBP	+ Reddy et al. (2006) [43]
BBP	+ Reddy et al. (2006) [43]
DnOP	+ Reddy et al. (2006) [43]
DEHP	+ Reddy et al. (2006) [43]; Nair et al. (2018) [47] - Cobellis et al. (2003) [44]; Kim et al. (2011) [51]
5oxo-MEHP	- Huang et al. (2010) [45]
5OH-MEHP	- Huang et al. (2010) [45]
MCPP	- Louis et al. (2013) [48]; Zhang et al. (2021) [52]
MCMHP	+ Louis et al. (2013) [48]
MNP	- Zhang et al. (2021) [52]
MOP	- Fernandez et al. (2019) [42]; Zhang et al. (2021) [52]
MNM	- Zhang et al. (2021) [52]
**Bisphenol A**	+ Upson (2014) [60]; Peinado et al. (2020) [61]; Rashidi et al. (2017) [62]; Simonelli et al. (2016) [63]; Wen et al. (2020) [65] - Fernandez et al. (2019) [42]; Louis et al. (2013) [48]; Cobellis et al. (2009) [64]; Itoh et al. (2007) [66]
**Parabens:** MeP	+ Peinado et al. (2020) [72]
EtP	- Peinado et al. (2020) [72]
PrP	- Peinado et al. (2020) [72]
BuP	- Peinado et al. (2020) [72]
**Benophenones:** BP-1	+ Peinado et al. (2020) [72]
BP-3	+ Peinado et al. (2020) [72]
4-OH-BP	- Peinado et al. (2020) [72]; Kunisue et al. (2012) [76]
2OH-4MeO-BP	- Kunisue et al. (2012) [76]
2,4OH-BP	- Kunisue et al. (2012) [76]
**Non persistent pesticides:** IMPY	+ Li et al. (2020) [87]
MDA	- Li et al. (2020) [87]
PNP	- Li et al. (2020) [87]
TCPY	+ Li et al. (2020) [87]
2,4-D	- Li et al. (2020) [87]
2,4,5-T	- Li et al. (2020) [87]
3-PBA	- Li et al. (2020) [87]
4F-3PBA	- Li et al. (2020) [87]
trans-DCCA	- Li et al. (2020) [87]
cis-DCCA	- Li et al. (2020) [87]
cis-DBCA	- Li et al. (2020) [87]

+ observed statistically significant effect; - no observed statistically significant effect.

## Data Availability

Not applicable.
